# Nutritional content of selected species of tropical eggplant fruit (*Solanum spp*) diet Attenuates hepatic inflammation in high‐fat fed male Wistar rats induced with streptozotocin

**DOI:** 10.1002/fsn3.811

**Published:** 2018-11-19

**Authors:** Esther Emem Nwanna, Emmanuel O. Ibukun, Ganiyu Oboh

**Affiliations:** ^1^ Department of Biochemistry Federal University of Technology Akure Nigeria

**Keywords:** glycemic index, hypolipidemia, nephrotoxicity, nutritional output, streptozotocin, Tropical eggplant

## Abstract

Tropical *Solanum* species contains a high level of phenolic acids and flavonoids, which were found to inhibit some key enzymes associated with the incidence of type 2 diabetes in in vitro and in vivo models based on earlier studies. This study was further designed to compare the nutritional properties, glycemic index, and hypolipidemic and the antioxidant effects of three species of tropical eggplant fruit (*Solanum kumba*,* Solanum aethiopicum,* and *Solanum gilo*) diet on streptozotocin (STZ)‐induced nephrotoxicity in male Wistar rats. The animal model was subjected to high‐fat diet prior to interperitional administration of streptozotocin (35 mg/kg wt); thereafter, the rats were given supplemented eggplant fruit diet, which lasted for 14 days. Determination of lipid content [triglycerides (TG), low‐density lipoproteins (LDLs), high‐density lipoproteins (HDLs), and total cholesterol (TC)], was assessed, while the liver biomarker enzymes alanine aminotransferase (ALT) alkaline phosphatase (ALP) and aspartate aminotransferase (AST), also endogenous enzymes such as superoxide dismutase (SOD) and catalase (CAT), were determined. Histopathological assessment of inflammation was carried out on kidney while the blood urea nitrogen (BUN), uric acid, and creatinine level on the kidney function were determined. The results showed that the groups with supplemented eggplant diet had significant (*p* < 0.05) reduction in lipid profile, decreased leakages of the liver, and kidney function enzymes while there was restoration of depleted endogenous antioxidant enzymes. The inflammatory cells and fat deposit from the histopathological view were reduced. However, *S. kumba* had the best nutritional output.

## 1 INTRODUCTION

Diabetes specifically (type II) has been typified as insulin resistance in which the major organs, liver, adipose and muscle tissues, are not quick to react to the action of insulin, as well as destruction of beta cells of islets of Langerhans which compromised the secretion of insulin produced (Reaven, 1995). Non‐insulin‐dependent diabetes mellitus and its complications such as myocardial infarction, hypercholesterolemia, atherosclerosis, hypertension, retinopathy, neuropathy (Ghoul, 2012) are increased if not properly managed due to various mechanisms such as oxidation of glucose and interaction of sugar molecules to proteins or lipids which have caused elevated blood glucose, and these have led to increased level of generation of reactive oxygen. Increased oxidative stress invariably increased the production of reactive oxygen species that is known as free radicals. These radicals are involved in the oxidation of protein in cellular structures, also in the oxidation of lipids which have led to cell injury associated with the pathology of vascular disease resulting in poor health and death in type II diabetes (Ghoul, 2012). Different reports have emphasized on natural products such as medicinal plants or plant fruits (Anosike, Abonyi, & Ubaka, 2011) and vegetables, based on folklore medicine to treat or manage diabetes mellitus (DM) (Nwanna et al., 2014). The use of natural products is pharmacologically active, not costly with little or minimal reactions as compared to other synthetic hypoglycemic agents. Fruits and vegetables have served a therapeutic role in different metabolic syndrome such as obesity, diabetes (Suhaila, 2014).

Eggplant commonly called garden egg or scarlet eggplants in West Africa are part of folklore remedy, which is used to curb elevated blood sugar and increased weight gain in Nigeria. According to American Diabetes Association (American Diabetes Association, 2009) and National Diabetes Education Programme of National Institute of Health, at Mayo Clinic, USA (Centres for Disease Control and Prevention (CDC), 1998), it was recommended that eggplant is a good source of plant food, which can be used to manage degenerative conditions. In addition, in vitro studies done by Kwon and his research group in 2008 on *Solanum melongena* on enzymes associated with diabetes confirmed this.

The tropical species of eggplant such as *Solanum kumba*,* Solanum aethiopicum*, and *Solanum gilo* are commonly found in all season in Nigeria, West Africa. They are consumed daily in different forms such as in soup and stew, eaten raw either used as fruits or vegetables. There is a need to scientifically explore these commonly found eggplant indigenous to sub‐Saharan Africa (Anosike et al., 2011; Nagaoka, Watanabe, Sakata, & Yoshihara, 2001). Nwanna et al. (2014) and Nwanna, Ibukun, and Oboh (2016) reported on the in vitro and the in vivo activities of these eggplant species in the management of type 2 diabetes. However, this present study further determines the effect of this eggplant diet supplementation on STZ‐induced nephrotoxicity in male Wistar rats and also assesses their hypolipidemia and antioxidant properties as well as the nutritional and glycemic index.

## 2 MATERIALS AND METHODS

### 2.1 Sample collection

Matured fruits of three eggplant species (*S. gilo, S. aethiopicum,* and *S. kumba)* were collected from the school farm in Ondo state, Nigeria. They were authenticated in the Department of Crop Soil and Pest of the Federal University of Technology Ondo state by Mr Omomoh B.E. The eggplant were cut into tiny slices, oven‐dried at 45°C, and grounded into flour and stored in an airtight container for experimental use.

The reagents and chemicals were purchased from Sigma‐Aldrich chemical company, the USA. In addition, some kits were ordered from Randox Laboratories.

### 2.2 Determination of proximate composition of eggplant species

Proximate analysis was carried out on the eggplant samples according to the method of Association of Official Analytical Chemists (AOAC) (2005) in order to determine the percentage of composition of protein, fat, fiber, ash, and the total carbohydrate.

### 2.3 Starch and sugar determination

The method of Onitilo, Sanni, Daniel, Maziya‐Dixon, and Dixon (2007) was used to determine the sugar and starch content of the sample. Hot ethanol 80% was used to digest 0.02 g of the sample, which was centrifuged at 2,000 *g* for 10 min. The supernatant was aspirated and used to determine the free sugar, while the residue was used to determine the start content according to the protocol. Glucose was used as the standard.

### 2.4 Determination of glycemic index

According to the method of Brounds et al. (2005), 25 mg of the eggplant powdered sample was weighed into a beaker, which was mixed with 5 ml of the stomach solution potassium chloride buffer at 1.5 pH and allowed in a shaker bath for 60 min at 40°C. It was then diluted with phosphate buffer pH 6.9 before the addition of 2.5 ml α‐amylase solution and allowed to stay at 37°C for 10 min. Thereafter, 200 μl of the digested sample was poured into test tube at 30‐min interval (0, 30, 60, 90, 120, 150, and 180) min. The aliquots were boiled for 15 min before addition of 500 μl sodium acetate pH 4.75 followed by 5 μl of the α‐glucosidase solution and then incubated for 45 min at 60°C. Two hundred microliters of DNSA solution was added and incubated for 5 min at 100°C; this was followed by an addition of 2 ml distilled water; and the mixture undergoes centrifugation at 3,000 *g* for 5 min. The supernatant was aspirated and read 540 nm. The sum of area under the curve for each sample was divided by the sum of area under the curve for standard glucose and multiplied by 100. The value obtained is the glycemic index.

### 2.5 Amylose content determination

According to the method of Goñi, Garcia‐Alonso, and Saura‐Calixto (1997) and Brounds et al. (2005), 1 ml of 95% ethanol and 9 ml of 1 M NaOH were added to 0.1 g of eggplant plant sample and standard (amylose). The mixture was heated for 10 min, 1 ml was taken and made up to 10 ml with distilled water, and 0.5 ml was taken out from the diluted sample while 0.1 ml of acetic acid and 0.2 ml of iodine solution were added. It was eventually made up to 10 ml using distilled water and was allowed to stay at room temperature for 10 min., after which vortexes and absorbance were taken at 620 nm. The results were calculated using the following formula: [Amylose of sample (%) = (Absorbance standard) % − Absorbance sample %)/Absorbance standard].

### 2.6 Amylopectin content determination

This was done using the method of Goñi et al. (1997). The percentage of amylose value of the amylose content determined was subtracted from 100%.

### 2.7 Experimental design

The methods of Oboh and Ogunruku (2010), Srinivasan, Viswanad, Asrat, Kaul, and Ramara (2005), and Nwanna et al. (2016) were used for the formulation of the food and the experimental design. Induction of rats to make them diabetic and the grouping were done according to the earlier studies reported by Nwanna et al. (2016). Various biochemical parameters were analyzed, the kidney was cut into small slices, and the method of Maynard and Downes (2014) was used to preserve it and later use for histopathological structure viewing.

Total cholesterol concentration was determined according to the method used by Adefegha and Oboh (2012). Triglyceride concentration was determined according to the method used by Oboh, Bello, and Ademosun (2014). High‐density lipoprotein cholesterol was determined using the method used by Adefegha et al. (2017). Low‐density lipoprotein cholesterol was determined using the method used by Oboh et al. (2014). Determination of malondialdehyde produced was done using the method of Ohkawa, Ohishi, and Yagi (1979). Plasma superoxide dismutase (SOD) activity was determined according to the method of Rietman and Frankel (1957). Catalase activity was determined using the method of Misra and Fridovich (1972). The methods of Beers and Sizer (1952) were used to determine the plasma transaminases such as alkaline phosphatase, ALP, aspartate aminotransferase, AST, and alanine aminotransferase, ALT. Protein content in all the tissues used was determined using the method of Lowry, Rosebrough, Farr, and Randall (1951).

### 2.8 Statistical analysis

The results of replicate readings were presented as means of standard deviation ± *SD*. Tukey's test and one‐way analysis of variance were used with GraphPad Prism version 7 software. A significant difference was taken to be *p* < 0.05 (Zar (1984)).

## 3 RESULTS

**Figure 1:**
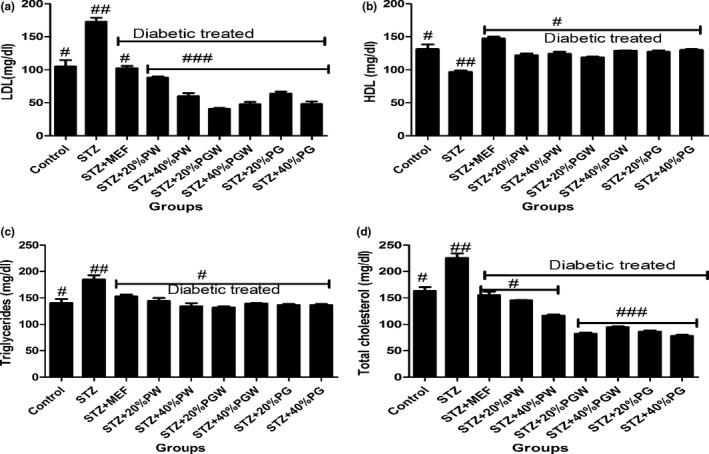
(a) Effect of eggplant supplemented diet on the low‐density lipoproteins level in the diabetic rats. Values represent mean ± standard deviation (*n* = 6). Bars with the same symbol are not significantly different (*p* > 0.05). There is significant difference (*p* < 0.05) in the bar with different symbol. MEF: metformin; PW:* Solanum gilo;* PGW:* Solanum aethiopicum;* PG:* Solanum kumba*. (b) Effect of eggplant supplemented diet on the high‐density lipoproteins level in the diabetic rats. Values represent mean ± standard deviation (*n* = 6). Bars with the same symbol are not significantly different (*p* < 0.05). There is significant difference (*p* < 0.05) in the bar with different symbol. MEF: metformin; PW:* Solanum gilo;* PGW:* Solanum aethiopicum;* PG:* Solanum kumba*. (c) Effect of eggplant supplemented diet on triglycerides level in the diabetic rats. Values represent mean ± standard deviation (*n* = 6). Bars with the same symbol are not significantly different (*p* < 0.05).). There is significant difference (*p* < 0.05) in the bar with different symbol. MEF: metformin; PW:* Solanum gilo;* PGW:* Solanum aethiopicum;* PG:* Solanum kumba*. (d) Effect of eggplant supplemented diet on total cholesterol level in the diabetic rats. Values represent mean ± standard deviation (*n* = 6). Bars with the same symbol are not significantly different (*p *< 0.05).). There is significant difference (*p* < 0.05) in the bar with different symbol. MEF: metformin; PW:* Solanum gilo;* PGW : *Solanum aethiopicum;* PG:* Solanum kumba*

**Figure 2:**
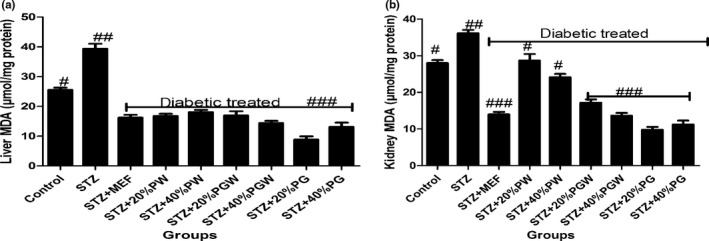
(a) Effect of eggplant supplemented diet on liver malondialdehyde (MDA) level in the diabetic rats. Values represent mean ± standard deviation (n = 6). Bars with the same symbol are not significantly different (*p *< 0.05). There is significant difference (*p* < 0.05) in the bar with different symbol. MEF: metformin; PW:* Solanum gilo;* PGW:* Solanum aethiopicum;* PG:* Solanum kumba*. (b) Effect of eggplant supplemented diet on kidney malondialdehyde (MDA) level in the diabetic rats. Values represent mean ± standard deviation (*n *= 6). Bars with the same symbol are not significantly different (*p* < 0.05). There is significant difference (*p* < 0.05) in the bar with different symbol. MEF: metformin; PW:* Solanum gilo;* PGW:* Solanum aethiopicum;* PG:* Solanum kumb*

**Figure 3:**
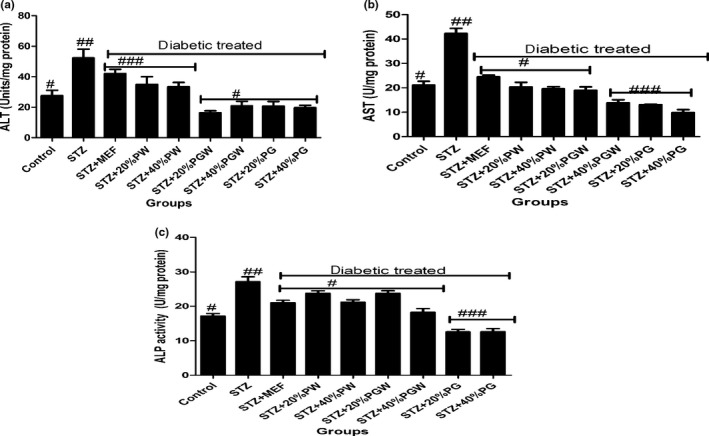
(a) Effect of eggplant supplemented diet on liver marker enzyme alanine aminotransferase (ALT) level in the diabetic rats. Values represent mean ± standard deviation (*n *= 6). Bars with the same symbol are not significantly different (*p* < 0.05). There is significant difference (*p* < 0.05) in the bar with different symbol. MEF: metformin; PW:* Solanum gilo;* PGW:* Solanum aethiopicum;* PG:* Solanum kumba*. (b) Effect of eggplant supplemented diet on liver marker enzyme aspartate aminotransaminase (AST) level in the diabetic rats. Values represent mean ± standard deviation (*n *= 6). Bars with the same symbol are not significantly different (*p* < 0.05). There is significant difference (*p* < 0.05) in the bar with different symbol. MEF: metformin; PW:* Solanum gilo;* PGW:* Solanum aethiopicum;* PG:* Solanum kumba*.(c) Effect of eggplant supplemented diet on liver marker enzyme alkaline phosphatase level in the diabetic rats. Values represent mean ± standard deviation (*n *= 6). Bars with the same symbol are not significantly different (*p* < 0.05). There is significant difference (*p* < 0.05) in the bar with different symbol. MEF: metformin; PW:* Solanum gilo;* PGW:* Solanum aethiopicum;* PG:* Solanum kumba*

**Figure 4:**
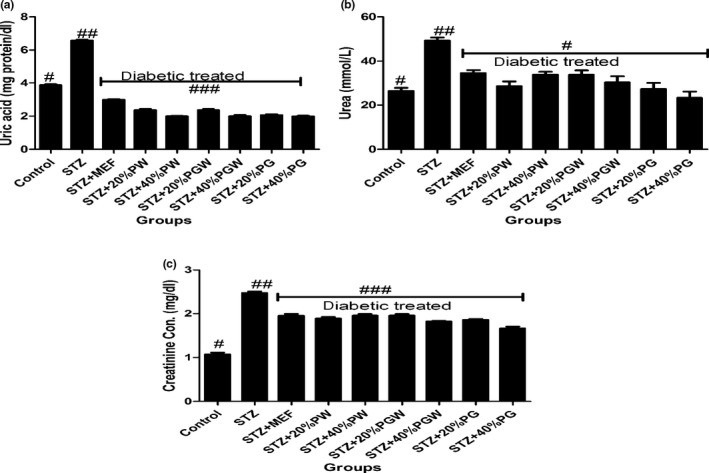
(a) Effect of eggplant supplemented diet on kidney uric acid level in the diabetic rats. Values represent mean ± standard deviation (*n *= 6). Bars with the same symbol are not significantly different (*p* < 0.05).). There is significant difference (*p* < 0.05) in the bar with different symbol. MEF: metformin; PW:* Solanum gilo;* PGW:* Solanum aethiopicum;* PG:* Solanum kumba*. (b) Effect of eggplant supplemented diets on kidney urea level in the diabetic rats. Values represent mean ± standard deviation (*n *= 6). Bars with the same symbol are not significantly different (*p* < 0.05). There is significant difference (*p* < 0.05) in the bar with different symbol. MEF: metformin; PW:* Solanum gilo;* PGW:* Solanum aethiopicum;* PG:* Solanum kumba*. (c) Effect of eggplant supplemented diet on kidney creatinine level in the diabetic rats. Values represent mean ± standard deviation (*n *= 6). Bars with the same symbol are not significantly different (*p* < 0.05). There is significant difference (*p* < 0.05) in the bar with different symbol. MEF: metformin; PW:* Solanum gilo;* PGW:* Solanum aethiopicum;* PG:* Solanum kumba*

**Figure 5:**
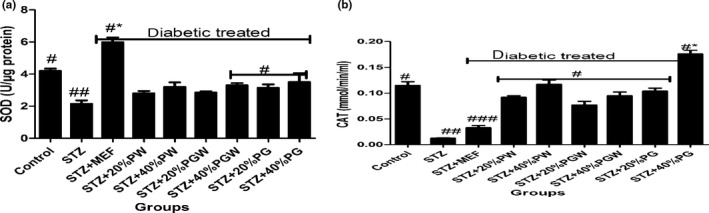
(a) Effect of eggplant supplemented diet on plasma superoxide dismutase (SOD) level in the diabetic rats. Values represent mean ± standard deviation (*n *= 6). Bars with the same symbol are not significantly different (*p* < 0.05).There is significant difference (*p* < 0.05) in the bar with different symbol. MEF: metformin; PW:* Solanum gilo;* PGW:* Solanum aethiopicum;* PG:* Solanum kumba*. (b) Effect of eggplant supplemented diet on plasma catalase level in the diabetic rats. Values represent mean ± standard deviation (*n *= 6). Bars with the same symbol are not significantly different (*p* < 0.05). There is significant difference (*p* < 0.05) in the bar with different symbol. MEF: metformin; PW:* Solanum gilo;* PGW:* Solanum aethiopicum;* PG:* Solanum kumba*

**Plate 1:**
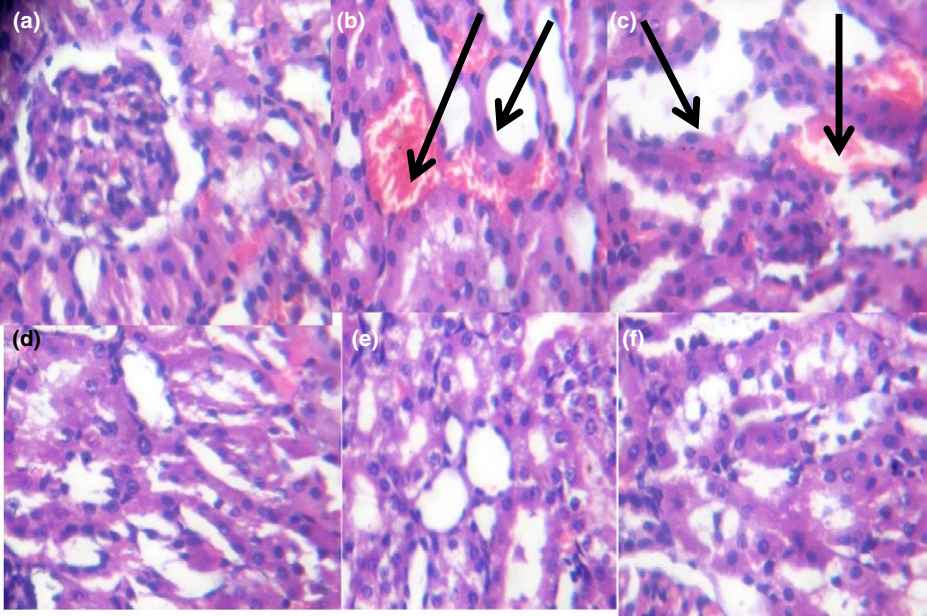
The effect of eggplant diet on the kidney. Histopathology view at *400. (a) Control rat revealed mild congestion of vessels, there is fatty infiltration of cortex, the glomeruli appear normal, and there is no evidence of effacement of the glomerular basement membrane. (b) Diabetic rat without treatment revealed focal area of interstitial fatty infiltration with inflammation (Black arrow) and congestion of vessels. (c) Diabetic rat given treatment with metformin revealed mild interstitial infiltration with inflammation (black arrow) and congestion of vessels with fatty infiltration of cortex. (d) Diabetic rat given treatment with 40% *S. gilo* revealed mild congestion with fatty infiltration of cortex, the glomeruli appear normal, and there is no evidence of effacement of the glomerular basement membrane. (e) Diabetic rat given treatment with 40% *S. aethiopicum* revealed mild congestion of vessels with less fatty infiltration of cortex, the glomeruli appear normal, and there is no evidence of effacement of the glomerular basement membrane. (f) Diabetic rat given treatment with 40% *S. kumba* revealed mild congestion of vessels, with less fatty infiltration of cortex, the glomeruli appear normal, and there is no evidence of effacement of the glomerular basement membrane

Tropical eggplant proximate composition as shown in Table 1 revealed that the eggplant species had moderate content of carbohydrate (61.78%‐70.06%), high fiber (7.46%‐9.97%), low fat (11.05‐14.675) low protein (5.04%‐5.75%), and low ash (5.68%‐8.44%) contents. Table 2 depicts the amylopectin between 30.22 and 3.64 g/100 g, while amylose ranges between 4.96 and 9.93 g/100 g. PG had significantly highest amylopectin values than PGW and PW. Table 3 shows the total sugar content ranges between 8.19 and 12.24 g/100 g), while the starch content ranges between 31.07 and 37 g per 100 g. The glycemic index is presented in Table 4, and it ranges between 30.16% and 38.65%. All the eggplant had low glycemic index below 50% (Table 5). Table 6 reveals average feed intake of the animal model with no significant (*p* > 0.05) difference in all groups. Table 7 reveals changes in average body weight (g). The positive control group had (26.24%) increased body weight gain, and the negative control (diabetic) group had reduced body weight while the diabetic rats fed with (20% and 40%) supplemented diet of the eggplant that had increased in body weight from 20.36% to 24.11%, respectively. Figure 1a–d reveals the triglyceride, total cholesterol, high‐density lipoprotein, and low‐density lipoprotein levels of the diabetic and nondiabetic model. The positive control group had increased level of triglycerides, total cholesterol, and low‐density lipoproteins while there was decreased high‐density lipoprotein level when compared with the STZ‐induced group. However, the groups induced with STZ and treated with metformin and eggplant diet had lower level of triglycerides, total cholesterol, and low‐density lipoproteins while there was increased high‐density lipoproteins. The difference was significant (*p* < 0.05) when compared to the negative control, but there was no difference between the treatment groups relative to metformin; but as observed, 40% PG eggplant diet was more effective than the 20%. The trend of the results followed the same vein for the malondialdehyde (MDA) produced as a result of the induction with STZ and how the treatment was able to combat it (Figure 2a,b). Also, Figure 3a‐c shows the effect of the STZ induction and treatment diet liver function such as ALT, AST, and ALP enzymatic activities was elevated but was reduced as a result of the eggplant treatment; these followed the same trend of earlier reports. Figure 4a‐c shows the histopathology results carried out on the kidney. The uric acid level, creatinine, and the blood urea nitrogen were elevated for the induced group with STZ without treatment, but the eggplant treatment diet was able to ameliorate the effect of the STZ and reduced the leakages significantly (*p* < 0.05). The endogenous antioxidant assess in this experiment catalase (CAT) and SOD was compromised; however, treatment with eggplant supplemented diet was able to manage as well as reduce the effect of the free radicals generated as a result of the STZ induction. Although metformin‐treated group (control drug) was able to ameliorate the effect better than the eggplant treatment group for SOD, eggplant diet elevated CAT especially *S. kumba* than *S. gilo* and *S. aethiopicum* as seen in Figure 5a,b. Plate 1a–f reveals the histopathology for kidney at 400 magnification from each of the study groups. Plate 1a is the control group, which shows mild congestion of the vessels which is focal, there is fatty infiltration of the cortex, the glomeruli appear normal, and there is no evidence of effacement of the glomerular basement membrane. Plate 1b is the induced group with STZ, which shows area of interstitial infiltration by inflammation as well as congestion of vessels, and there is also more fatty infiltration of the cortex. Plate 1c is the metformin‐treated group, which shows congestion of vessels as well as less fatty infiltration of cortex with mild inflammation, the glomeruli appear normal, and there is no evidence of effacement of the glomerular basement membrane. Plate 1d is the diabetic treated with *S. gilo,* which shows mild congestion of vessels with less fatty infiltration of the cortex, the glomeruli appear normal, and there is no evidence of effacement of the glomerular basement membrane. Plate 1e and f which is the diabetic treated with *S. aethiopicum* and *S. kumba* shows mild congestion of vessels, more less fatty infiltration of the cortex, the glomeruli appear normal, and there is no evidence of effacement of the glomerular basement membrane. There were no inflammatory cells observed in the groups with eggplant supplemented diet. Eggplant was able to manage the effect of the STZ on the kidney better than the metformin‐treated group.

**Table 1:** Proximate composition of selected eggplant species

Sample	%Ash	% Fat	%CF	%CP	% Carb
PGW	7.59 ± 1.2^a^	11.55 ± 1.89^a^	8.98 ± 2.55^a^	5.04 ± 1.70^a^	66.84 ± 4.50^a^
PG	8.44 ± 1.1^b^	14.67 ± 2.54^b^	9.97 ± 1.39^b^	5.14 ± 1.30^a^	61.78 ± 3.77^b^
PW	5.68 ± 1.3^c^	11.05 ± 1.99^a^	7.46 ± 2.21^c^	5.75 ± 1.40^b^	70.06 ± 5.12^c^

*Notes*

Values represent mean ± standard replicate readings.

PGW: *Solanum aethiopicum;* PG: *Solanum kumba;* PW: *Solanum gilo*; CF: crude fiber; CP: crude protein; Carb: carbohydrate.

The values with different letter are significantly different (*p* < 0.05).

**Table 2:** Composition of amylose and amylopectin (g/100 g) of the eggplant species

Parameter	Amylose	Amylopectin
PGW	4.96 ± 1.02^b^	33.64 ± 1.27^b^
PG	9.93 ± 2.05^a^	30.22 ± 2.01^a^
PW	4.96 ± 1.01^b^	32.69 ± 1.20^b^

*Notes*

Data represent means of duplicate determinations. Values with the same letter along the same column are not significantly different (*p* < 0.05).

PGW: *Solanum aethiopicum;* PG: *Solanum kumba;* PW: *Solanum gilo*.

**Table 3:** Composition of total sugar and starch (g/100 g) of the eggplant species

Parameter	Total sugar	Starch
PGW	8.19 ± 1.12^a^	31.07 ± 2.94^a^
PG	10.35 ± 1.99^b^	34.83 ± 2.91^b^
PW	12.24 ± 2.01^c^	37.66 ± 2.23^c^

*Notes*

Data represent means of duplicate determinations. Values with the same letter along the same column are not significantly different (*p* < 0.05).

PGW: *Solanum aethiopicum;* PG: *Solanum kumba;* PW: *Solanum gilo*.

**Table 4:** Glycemic index of the eggplant species

Parameter	Glycemic index (%)
PGW	30.16 ± 2.55^a^
PG	30.60 ± 2.57^a^
PW	38.65 ± 3.76^b^

*Notes*

Data represent means of duplicate determinations.Values with the same letter along the same column are not significantly different (*p* < 0.05).

PGW: *Solanum aethiopicum;* PG: *Solanum kumba;* PW: *Solanum gilo*.

**Table 5:** (a) High‐fat diet formulation for basal and supplemented diet for control and test groups (g/kg). (b) Diet formulation for basal and supplemented diet for control and test groups (g/kg)

Content	Group 1	Group 2	Group 3	Group 4	Group 5	Group 6	Group 7	Group 8	Group 9
(a)									
S/milk	444	444	444	416	389	414	381	422	397
Corn flour	416	416	416	244	71	246	79	238	63
Lard	300	300	300	300	300	300	300	300	300
Premix	40	40	40	40	40	40	40	40	40
Sample	‐	‐	‐	200	400	200	400	200	400

(b)									
S/milk	444	444	444	416	389	414	381	422	397
Corn flour	416	416	416	244	71	246	79	238	63
Oil	100	100	100	100	100	100	100	100	100
Premix	40	40	40	40	40	40	40	40	40
Sample	‐	‐	‐	200	400	200	400	200	400

*Notes*

Skimmed milk (S/milk) = 32% protein; the vitamin mixture (mg or IU/g) has the following composition: 3,200 IU vitamin A, 600 IU vitamin D3, 2.8 mg vitamin E, 0.6 mg vitamin K3, 0.8 mg vitamin B1, 1 mg vitamin B2, 6 mg niacin, 2.2 mg pantothenic acid, 0.8 mg vitamin B6, 0.004 mg vitamin B12, 0.2 mg folic acid, 0.1 mg biotin H2, 70 mg choline chloride, 0.08 mg cobalt, 1.2 mg copper, 0.4 mg iodine, 8.4 mg iron, 16 mg manganese, 0.08 mg selenium, 12.4 mg zinc, 0.5 mg antioxidant.

Group 1 (control): Normal rats received citrate buffer (pH 4.5) (1 ml/kg, i.p.) and fed with basal diet only. Group 2 (STZ only): Diabetic rats fed with basal diet only. Group 3 (STZ and metformin): Diabetic rats (35 mg/kg body wt) received 100 mg/kg i.p metformin orally/per day and fed with basal diet only. Group 4 (STZ+20% PW): Diabetic rats fed with basal diet supplemented with 20% *Solanum gilo*. Group 5 (STZ+40% PW): Diabetic rats fed with diet supplemented with 40% *S. gilo*. Group 6 (STZ+20% PGW): Diabetic rats fed with diet supplemented with 20% *Solanum aethiopicum*. Group 7 (STZ+40% PGW): Diabetic rats fed with diet supplemented with 40% *S. aethiopicum*. Group 8 (STZ+20% PG): Diabetic rats fed with diet supplemented with 20% *Solanum kumba*. Group 9 (STZ+40% PG): Diabetic rats fed with diet supplemented with 40% *S. kumba*.

**Table 6:** Average food intake of rats fed eggplant supplemented diet

Treatment group	Average feed intake (g/rat/day)
Control (basal diet)	18.13 ± 2.27^a^
STZ (diabetic control)	19.89 ± 3.11^a^
STZ+ metformin (control drug)	18.43 ± 2.15^a^
STZ+20% PW	17.55 ± 1.01^a^
STZ+40% PW	17.89 ± 1.11^a^
STZ+20% PGW	17.50 ± 1.17^a^
STZ+40% PGW	18.34 ± 1.05^a^
STZ+20% PG	18.76 ± 2.05^a^
STZ+40% PG	18.67 ± 2.12^a^

*Notes*

The effect of diet supplemented with *Solanum* species on blood glucose level in HFD/low‐dose STZ‐induced diabetic rats (35 mg/kg bwt). Values represent mean ± standard deviation (*n* = 6). There was no significant difference (*p* < 0.05) in the average feed intake.

Key: group 1: normal rats received citrate buffer (pH 4.5) (1 ml/kg i.p) and fed with basal diet only, group 2: Diabetic rats fed with basal diet only, group 3: Diabetic rats received 100 mg/kg orally metformin and fed with basal diet only, group 4: Diabetic rats fed with basal diet supplemented with 20% *Solanum gilo* (PW), group 5: Diabetic rats fed with diet supplemented with 40% *S. gilo* (PW), group 6: Diabetic rats fed with diet supplemented with 20% *Solanum aethiopicum* (PGW), group 7: Diabetic rats fed with diet supplemented with 40% *S. aethiopicum* (PGW), group 8: Diabetic rats fed with diet supplemented with 20% *Solanum kumba* (PG), and group 9: Diabetic rats fed with diet supplemented with 40% *S. kumba* (PG).

The values with different letter are significantly different (*p* < 0.05).

**Table 7:** Change in average body weight (g) of diabetic rats fed eggplant supplemented diet

Treatment group	%Weight gain
Control (basal diet)	26.24^a^
STZ (diabetic control)	−28.29^b^
STZ+ metformin (control drug)	24.78^c^
STZ+20% PW	20.36^d^
STZ+40% PW	21.97^d^
STZ+20% PGW	23.08^d^
STZ+40% PGW	23.12^d^
STZ+20% PG	22.18^d^
STZ+40% PG	24.11^d^

*Notes*

The effect of diet supplemented with *Solanum* species on blood glucose level in HFD/low‐dose STZ‐induced diabetic rats(35 mg/kg bwt). Values represent mean ± standard deviation (*n* = 6). Values with the same superscript letters are not significantly different (*p* < 0.05).

Key: group 1: Normal rats received citrate buffer (pH 4.5) (1 ml/kg i.p) and fed with basal diet only, group 2: Diabetic rats fed with basal diet only, group 3: Diabetic rats received 100 mg/kg orally metformin and fed with basal diet only, group 4: Diabetic rats fed with basal diet supplemented with 20% *Solanum gilo* (PW), group 5: Diabetic rats fed with diet supplemented with 40% *S. gilo* (PW), group 6: Diabetic rats fed with diet supplemented with 20% *Solanum aethiopicum* (PGW),group 7: Diabetic rats fed with diet supplemented with 40% *S. aethiopicum* (PGW), group 8: Diabetic rats fed with diet supplemented with 20% *Solanum kumba* (PG), and group 9: Diabetic rats fed with diet supplemented with 40% *S. kumba* (PG).

## 4 DISCUSSION

Proximate analysis was done to determine the major constituents in the eggplant species such as carbohydrate, protein, fat, ash, fiber content; the eggplant had low carbohydrate content, low fat, and high fiber content when compared with the studies of Irondi, Oboh and Akindahunsi (2016) on *Mangifera indica* kernel, which could be due to the polyphenol constituents as reported earlier (Nwanna, Ibukun, & Oboh, 2013). Reports from Jenkins et al. (1981) showed that glucose responses are products from carbohydrate foods consumed, the types of food, and the extent to which it was processed. The slower the degradation of carbohydrate‐containing foods, the lower the level of glucose in the blood, which is relative to the value of glycemic index; thus, the food source or that particular diet can be termed to have low GI. Therefore, GI is the ability to rank carbohydrate‐containing foods associated with the glucose level in the blood after consumption on a scale of 100% with 100 representing pure glucose. The benefits of assessing the glycemic index of foodstuffs serve as glucose control, control of insulin demands, and low concentration of lipids in the blood, which are invariably linked to the control and/or prevention of diseases such as cardiovascular disease, diabetes, and hypertension. Previous studies on carbohydrates, “isomaltulose,” by Ines (2010) and Fitzgerald et al. (2011) on “rice” revealed that low glycemic diet with low insulinemic diet reduces diseases and the level of susceptibility to different noncommunicable diseases such as diabetes and hypertension. Phytochemicals from eggplant have received much attention due to their phenolic/flavonoids compounds (Huang et al., 2013). This present study was carried out to further explore the function of these indigenous species of eggplant *S. gilo*,* kumba,* and *aethiopicum* of tropical origin commonly consumed by the people in West Africa. The findings revealed low glycemic index for these species, which is closely related to the findings of Chan, Patricia, Suhaimi, Yasir, and Tek (2014) on “red seaweed” because of their characteristics of polyphenol content. The low glycemic index, based on the amount of sugar and starch content, and its percentage of amylose and amylopectin 1:3 for *S. aethiopicum* and 1:8 for the *S. kumba* and *S. gilo* were observed. These findings are proportional to their carbohydrate and fiber content from the analysis. Hettiarachchi, Jiffry, Wickramasinghe, and Fernando (2001) and Jeya et al. (2007) categorized foods within the content of low glycemic index (<55), medium glycemic index (56‐69), and high glycemic index (>70). This tropical eggplant falls below 55, a low GI diet, which is encouraged for good health. Hyperlipidemia is a known contributing risk factor, which has led to high accumulation and deposits of fats in the vessels (Mengesha, 2006), and also predisposed the system to altered metabolism of lipids, which have contributed to the incidence of atherosclerosis in diabetes mellitus. The induction of the animal model with streptozotocin invariably increases the accumulation and saturation of glucose due to the destruction of pancreatic β‐cells with much lower healthy cells (Kamtchoung et al. 1998); eventually, the glucose has to be stored as fat in different tissues. This causes disruption in lipid profile parameters such as total cholesterol, low‐density lipoproteins, and triglycerides and a decline in high‐density lipoproteins. But the eggplant diet was able to ameliorate the effect of streptozotocin due to the various phenolic acid and flavonoid presence when compared with metformin treatment. In the same vein, the destruction of the pancreatic β‐cells also led to loss of body weight due to increased degradation of structural proteins, which contributes to the body mass index. The body weight was restored with the eggplant supplemented diet compared to the untreated group because of the effect of the antioxidant properties of the polyphenols in the eggplant (Nwanna et al., 2014); however, there was no significant change in the daily feed intake (*p* > 0.05).

The elevated level of antioxidant enzymes catalase and superoxide dismutase in the study revealed the significant role played by polyphenols in the eggplant diet in diabetic subject as against the ones without treatment. The phenolic and flavonoid presence was able to combat the effect of reactive oxygen species generated during the excessive metabolism of glucose; the SOD decreased in all treated groups while CAT activity was elevated, the reason is because the SOD was already saturated in its action while CAT took over the action in mopping off the remaining radicals produced.

Malondialdehyde produced in the diabetic group without treatment was more as observed in the kidney and liver tissues when compared to a normal healthy group and the diabetic groups treated with metformin and eggplant supplementation. This is because the antioxidant defense mechanism has been compromised but supplemented eggplant diet/metformin was able to manage or treat the diabetic subjects, which invariably reduced the radical species generation during auto‐oxidation of glucose which could be due to the phenolic acids and flavonoids in the eggplant (Nwanna et al., 2016).

In disease investigation and diagnosis, ability to measure enzyme activity in tissue is paramount (Malomo, 2000). This measurement or the level of activity of this enzyme could give information concerning the organ or tissue wherever there is damage. The activities of ALP, AST, and ALT in the liver tissue are important in amino acid metabolism, which provide by‐products or intermediates for another pathway like gluconeogenesis (Malomo, 2000). However, there was a decline in these enzyme activities as a result of deleterious effect of STZ induction, but the eggplant diet reduced the effect of the STZ and restores the enzyme functionality. Epidemiological reports by Oksana, Marek, and Marian (2016) revealed that there is an association between consumption of phenolics from plant foods and combating of diseases because the phenolics have antioxidant effect as a result of the sparing effect of the hydroxyl groups.

Morphology and functional structure of the organs during the incidence of diabetes are normally altered according to what was reported by Sochar, Baquer, and Mclean (1985). The histopathological view of the kidney of the diabetic subjects without treatment revealed inflammatory cell infiltration when compared to the healthy control and the diabetic treated subject, although there was no significant lesion in the kidney when compared to the kidney of the ones that received treatment. In addition, the kidney of diabetic subject showed a mild increased in fats as a result of excess glucose in the blood, which has to be stored and could have to assess to the subcapsular area. But the diabetic model given supplemented eggplant diet had little or no inflammatory cells as well as fat deposit. This result confirms the report of Anosike, Onyechi, Lawrence, and Ezeanyika (2012) on the anti‐inflammatory activity of *S. aethiopicum*. It was the groups with 40% supplementation that was used for the histopathology view because 40% inclusion was better in their output in this study. The incidence of diabetic nephropathy showed elevated kidney markers like creatinine, uric acid, and blood urea nitrogen in the animal model due to the inability of the kidney to filter out these waste products of metabolism efficiently; the eggplant supplemented diet was able to reduce these products to restore the function of the kidney to normal as evidenced in the kidney enzyme activities. This report agrees with the study of Kalaivanan and Pugalendi (2011). During diabetes, there is overload and excess production of different by‐products during the process of different metabolism whether anabolism or catabolism such as glycogenolysis gluconeogenesis after glycolysis (Latner, 1985) but a better therapeutic source such as functional foodstuffs with a low glycemic index could manage these effects.

In the same vein, the inclusion of the tropical eggplant in the diet could restore and reverse the diabetic state of the diabetic model due to the presence of polyphenol and flavonoid constitutes as reported earlier by Nwanna et al. (2014, 2016) from the high‐performance liquid chromatogram, which depicts the individual phenolic acids and flavonoids. It was reported that the concentration of these constituents was more in *S. kumba,* which could have contributed to the observed activities.

## 5 CONCLUSION

Our findings suggest that tropical eggplant diet could have a holistic effect on various pathology as a result of its abundant polyphenol constituents and its low glycemic index, which could serve as therapeutic functional food that can be used to manage/control diabetes and diabetes‐related complications such as hepatic inflammation.

## CONFLICT OF INTEREST

The authors have no conflict of interest.

## ETHICAL APPROVAL

Approval was obtained from the relevant School's ethics committee responsible for the use of laboratory animals. The handling and use of the experimental animals was as approved by the Animal Ethics Committee of the School of Sciences, Federal University of Technology, Akure, Nigeria, with protocol reference SOS/14/02, and was in accordance with the NIH guide for the use and handling of experimental animals.

## Figures and Tables

**Plate A1:**
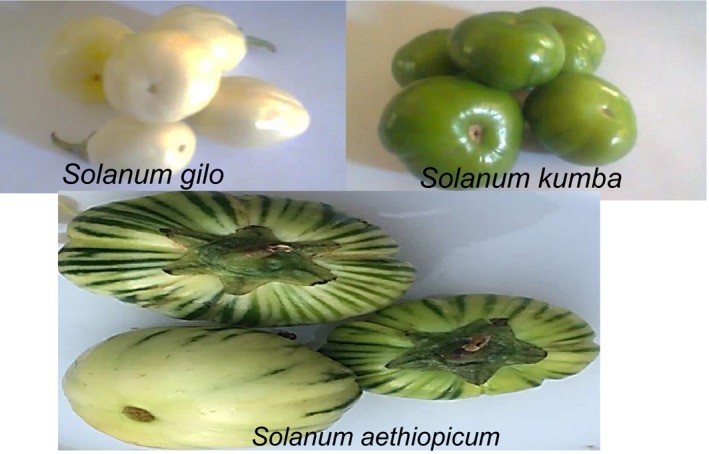
Caption of Selected Tropical Eggplant Species
